# Molecularly Imprinted Polymers Specific towards 4-Borono-L-phenylalanine—Synthesis Optimization, Theoretical Analysis, Morphology Investigation, Cytotoxicity, and Release Studies

**DOI:** 10.3390/polym15143149

**Published:** 2023-07-24

**Authors:** Emilia Balcer, Monika Sobiech, Joanna Giebułtowicz, Małgorzata Sochacka, Piotr Luliński

**Affiliations:** 1 Department of Drug Chemistry, Faculty of Pharmacy, Medical University of Warsaw, Banacha 1, 02-097 Warsaw, Poland; emilia.balcer@wum.edu.pl (E.B.); joanna.giebultowicz@wum.edu.pl (J.G.); malgorzata.bogucka@wum.edu.pl (M.S.); 2 Radiochemistry Team, Reactor Research Division, Nuclear Facilities Operations Department, National Centre for Nuclear Research, Sołtana 7, Świerk, 05-400 Otwock, Poland; 3 Department of Organic and Physical Chemistry, Faculty of Pharmacy, Medical University of Warsaw, Banacha 1, 02-097 Warsaw, Poland; monika.sobiech@wum.edu.pl

**Keywords:** 4-borono-L-phenylalanine, molecularly imprinted polymer, morphology, cytotoxicity, boron carrier

## Abstract

The aim of this study was to create molecularly imprinted polymers (MIPs) that are specific towards 4-borono-L-phenylalanine (BPA) to serve as boron compound carriers. The honeycomb-like MIPs were characterized in the matter of adsorption properties, morphology, structure, and cytotoxicity towards A549 and V79-4 cell lines. The honeycomb-like MIP composed from methacrylic acid and ethylene glycol dimethacrylate was characterized by a binding capacity of 330.4 ± 4.6 ng g^−1^ and an imprinting factor of 2.04, and its ordered, porous morphology was confirmed with scanning electron microscopy. The theoretical analysis revealed that the coexistence of different anionic forms of the analyte in basic solution might lower the binding capacity of the MIP towards BPA. The release profiles from the model phosphate buffer saline showed that only 0 to 4.81% of BPA was released from the MIP within the time frame of two hours, furthermore, the obtained material was considered non-cytotoxic towards tested cell lines. The results prove that MIPs can be considered as effective BPA delivery systems for biomedical applications and should be investigated in further studies.

## 1. Introduction

Boron neutron capture therapy (BNCT) is a type of cancer treatment that uses nuclear capture and fission reactions, occurring when the atoms of boron-10 (^10^B) are irradiated with neutrons from an external beam. The products of this reaction, α particles and lithium nuclei, in theory, kill only ^10^B-containing cells in the immediate vicinity of the reaction [1]. Thus, the effectiveness of BNCT is dependent on the selective delivery of ^10^B to the tumor site.

Providing the success of finding the appropriate boron carrier, BNCT has the potential to become not only an alternative treatment method but also a first-choice option in the case of inoperative or metastatic tumors. A significant advantage of BNCT in comparison to conventional methods, such as radio- or chemotherapy, is the possibility of limiting the exposition of healthy cells to an excessive dose of radiation due to the minimized interaction between the neutrons and the tissues with no deposited boron. Moreover, the nature of neutrons themselves allows the avoidance of damage that is caused by different types of ionizing radiation. This advantage is directly linked to the reduction in adverse effects of therapy, which further greatly influences the quality of life of the patients—an aspect that should be prioritized in every case. One of the biggest obstacles in the widespread application and further development of BNCT remains the challenge of providing an ideal boron carrier. Such a compound should be characterized by a low toxicity and high biocompatibility, high specificity to tumor cells, sufficient capacity for the delivery of 20–50 µg of boron per g of tumor tissue, concentration ratio in tumor-healthy tissues and tumor-blood greater than 3:1, high and fast elimination from blood and normal cells, and retention in a tumor long enough for the duration of irradiation [2]. Additionally, the compounds dedicated to brain tumors should have the ability to penetrate the blood–brain barrier.

Despite the fact that various boron carriers have been designed and described, at the moment, only two compounds are used in clinical trials, 4-borono-L-phenylalanine (BPA) and sodium mercaptoundecahydro-closo-dodecaborate (BSH) [3]. Even though BSH shows the advantage of having 12 boron atoms per molecule, its use is limited due to the passive diffusion mechanism that governs its transport, which results in insufficiently selective delivery [1]. Thus, most of the scientific interest is focused on BPA since it is delivered to cancerous cells using the mechanism of active uptake mediated by L-amino acid transporters (primarily LAT1), providing more efficient therapy. However, the BPA solubility in water at blood pH level is insufficiently low. For that purpose, various alternative modifications were proposed to tackle this problem, e.g., the complex of BPA with D-fructose [4,5,6].

Recently, boron-based polymers, such as organoboranes, borazine (polymers with conjugated boron heterocyclic systems), boron quinolates, derivatives of boronic acid and functionalized boronic ester polymers, carborane, or borane clusters containing polymers, have attracted scientific attention because of their effectiveness in penetrating cancerous tissues and the homogeneous delivery of the boron clusters into tumor cells [7]. A very promising solution was proposed by Nomoto et al. [8], where BPA was complexed with poly(vinyl alcohol) (PVA), which caused the formulation to be transported into tumor cells by LAT1-mediated endocytosis and thus slowed the unwanted efflux. The formed PVA–BPA complex demonstrated a higher accumulation and longer retention in the tumor than the fructose–BPA complex, significantly improving the therapeutic effect. The application of boron-based polymers looks like a promising alternative for a BNCT-dedicated compound, but some synthetic routes and post-polymerization modifications could hamper their utility [9]. 

Thus, one of interesting options is to use the molecularly imprinted polymers (MIPs) as the carriers of boron with the potential for further application in BNCT. The MIPs are a class of polymeric materials formed during a three-step synthetic process which includes creating a prepolymerization complex with selected functional monomer(s) involving non-covalent interactions or a chemical reaction, the polymerization process, and in the last step, the template removal from the polymeric matrix. Today, MIPs are considered to be a well-known class of materials that are used in separation science and in the detection of molecules, mostly due to their beneficial properties such as high selectivity, thermal and chemical stability, and reusability [10,11]. The facile preparation and capability for their integration with other compounds or systems to fabricate composite devices became additional advantages of MIPs, causing their broad utilization in the field of analytical chemistry [12,13]. The potential of MIPs as drug delivery devices was also investigated [14,15]. However, it should be emphasized that a MIP being an integrated part of the device gives it the opportunity to not only serve as the drug carriers (for example, in the treatment of cancer) but also to act as ligands that are able to recognize specific domains on the surface of cells (for instance, glycoproteins overexpressed in the cancer process) [16].

In this paper, we are interested in investigating the MIP with potential capability in the delivery of boron. In order to achieve this goal, in the synthesis optimization step, the theoretical analysis was employed to reduce the costs at the step of functional monomer selection. Moreover, a cavity of the MIP generated in silico allowed us to explain the interactions between the BPA and the polymer matrix to prove the specificity. Comprehensive morphological investigations of MIPs prepared from various cross-linking reagents were performed and compared to the results of the binding capacities. Biocompatibility studies were carried out, and the release of the target molecule was analyzed to verify the MIP’s potential as a boron carrier. It has to be emphasized that to the best of our knowledge, the MIP for boron-containing molecule delivery has not been designed. Thus, it constitutes as a novelty of our study. It has to be underlined that the presented results comprise a preliminary stage of the investigations related to MIP-based boron delivery devices.

## 2. Materials and Methods

### 2.1. Reagents and Standards

A template molecule, L-phenylalanine, and the target molecule, BPA, were purchased from Sigma-Aldrich (Steinheim, Germany). The functional monomer: methacrylic acid; the cross-linkers: ethylene glycol dimethacrylate (**1**), poly(ethylene) glycol dimethacrylate (**2**), trimethylolpropane trimethacrylate (**3**), triethylene glycol dimethacrylate (**4**), and divinylbenzene (**5**); and the silane components: tetraethoxysilane and 3-(trimethoxysilyl)propyl methacrylate (MPS) were purchased from Sigma-Aldrich (Steinheim, Germany). The solvents: acetonitrile, methanol, ethanol, propan-2-ol, toluene, acetone, glacial acetic acid, trifluoroacetic acid, hydrofluoric acid (48–50%), hydrochloric acid (36%), formic acid (98%), acetic acid, and ammonium hydroxide (25%), and salts: ammonium acetate and sodium hydroxide, were brought from POCh (Gliwice, Poland). The polymerization reaction initiator, 1,1′-azobiscyclohexanecarbonitrile, was from Fluka (Steinheim, Germany). The HPLC gradient-grade methanol, acetic acid, and acetonitrile were purchased from Merck (Darmstadt, Germany). Ultra-pure water, delivered from a Hydrolab HLP 5 system (Straszyn, Poland), was used to prepare the water solutions. 

The A549 (human non-small cell lung carcinoma) cell line was provided by the Department of Applied Toxicology, Faculty of Pharmacy, Medical University of Warsaw, and the V79-4 (Chinese hamster lung fibroblasts) cell line was provided by the Department of Environmental Health Science, Faculty of Pharmacy, Medical University of Warsaw. Dulbecco’s Modified Eagle Medium (DMEM), Kaighn’s Modification of Ham’s F-12 Medium (F12-K), streptomycin, penicillin, fetal bovine serum (FBS), and phosphate buffered saline (PBS) were purchased from Gibco (Paisley, United Kingdom). The 3-(4,5-dimethylthiazol-2-yl)-2,5-diphenyltetrazolium bromide (MTT) was brought from Sigma-Aldrich (Steinheim, Germany). 

### 2.2. Polymers

#### 2.2.1. Bulk Polymer

The radical bulk polymerization was performed to obtain a polymer from methacrylic acid and ethylene glycol dimethacrylate, coded as MIP**1_b_** (index b refers to bulk) in the presence of L-phenylalanine, acting as the template. The corresponding non-imprinted polymer, NIP**1_b_**, was prepared in the same way but without the template. The experimental amounts of reagents are provided in Table 1. Briefly, L-phenylalanine (0.35 mmol), methacrylic acid (2 mmol), and ethylene glycol dimethacrylate, were dissolved in a mixture of toluene, glacial acetic acid, and trifluoroacetic acid in a falcon-tube. The addition of glacial acetic acid and trifluoroacetic acid facilitates the dissolution of L-phenylalanine in the porogenic solvent of toluene [17]. Then, the polymerization initiator, 1,1′-azobiscyclohexanecarbonitrile, was added, and the homogeneous solution was purged with nitrogen for five minutes (min) before sealing the tube. Subsequently, the polymerization was carried out in a nitrogen atmosphere for 24 h at 88 °C. The bulk rigid polymer was then ground and wet-sieved into particles below 45 μm in diameter. Fine particles were separated by repeated decantation from acetone. The removal of L-phenylalanine was processed, using continuous extraction in a Soxhlet apparatus (24–36 h, 80 mL, methanol), followed by a washing sequence (in brackets, volumes of solvent per 10 mg of polymer are provided): methanol–1% aq. ammonium hydroxide (10 mL), methanol–1% aq. formic acid (10 mL), methanol–40 mmol L^−1^ aq. ammonium acetate (70:30, *v*/*v*, 10 mL), and 80% aq. methanol (1 mL). The template removal was monitored using liquid chromatography coupled with tandem mass spectrometry (LC-MS/MS). The particles were dried in a vacuum at room temperature. 

#### 2.2.2. Synthesis of Siloxane Particles 

The siloxane particles were synthesized according to Stöber method [18]. For this purpose, a solution of 12.5 mL of tetraethoxysilane in 200 mL of anhydrous ethanol was added to two-neck round-bottom flask. Then, a solution of 12 mL of 25% aq. ammonium hydroxide in 20 mL of ultra-pure water was added dropwise. The reaction mixture was stirred at 150 rpm for 2 h at room temperature. The white precipitate was collected by centrifugation (700 rpm, 45 min) and was washed three times with anhydrous ethanol. Next, the siloxane particles were functionalized with MPS. For this purpose, an amount of 3.9 g of siloxane particles was dispersed in 195 mL of anhydrous toluene in a round-bottom flask. Then, 19.5 mL of MPS was added, and mixture was stirred at 400 rpm at room temperature for 24 h. Obtained functionalized siloxane particles coded as SiO_2_@MPS were centrifuged (700 rpm, 30 min) and were washed three times with anhydrous toluene prior to drying. 

#### 2.2.3. Honeycomb-like Polymers

The radical polymerization was performed to obtain the honeycomb-like polymers from methacrylic acid (2 mmol) and various cross-linkers, coded as MIP**1_h_**–MIP**5_h_** (index h refers to honeycomb-like) in the presence of L-phenylalanine (0.35 mmol), acting as the template. The corresponding non-imprinted polymers, NIP**1_h_**–NIP**5_h_**, were prepared in the same way but without the template. The experimental amounts of the reagents (moles, masses, and volumes) used for the preparation of the different types of polymers are listed in Table 1.

Briefly, L-phenylalanine, methacrylic acid, and a selected cross-linker, were dissolved in a mixture of toluene, glacial acetic acid, and trifluoroacetic acid in a falcon-tube prior to purging with nitrogen for five min and transferring to another falcon-tube with separately weighted particles of SiO_2_@MPS. Next, the content (pre-polymerization mixture together with functionalized siloxane particles) was placed into the ultrasonic bath for 15 min. After, the initiator of the polymerization reaction was added, the falcon-tube was purged with nitrogen once again for five min, and then put into the oil bath for 24 h, keeping the temperature at 94 °C and allowing the polymerization reaction to proceed. Next, the polymer was removed from falcon-tube and was gently crushed to obtain irregular particles of ca 2–5 mm of diameter which were put into the solution of 2.5% aq. hydrofluoric acid, keeping the proportion of five–ten particles per 5 mL of the solution. The etching of siloxane lasted for 3 days. Then, the solution of 2.5% aq. hydrofluoric acid was removed, and the particles were transferred to deionized water (5 mL) for 1 day. Finally, the water was removed, and the particles were washed twice with methanol (10 mL). Next, a washing sequence was carried out to remove the template (in brackets, volumes of solvent per 10 mg of polymer are provided): methanol–1% aq. ammonium hydroxide (1.5 mL), methanol–1% aq. formic acid (1.5 mL), methanol–40 mmol L^−1^ aq. ammonium acetate (70:30, *v*/*v*, 1.5 mL), and 80% aq. methanol (1 mL). The sequence was repeated six times with intervals between the change of solution from 4 to 24 h. The template removal was monitored using LC-MS/MS. The particles were dried in a vacuum at room temperature.

### 2.3. Binding Studies

For binding studies, polypropylene tubes were filled with 10 mg of MIP**1_b_** and MIP**1_h_**–MIP**5_h_**, NIP**1_b_** and NIP**1_h_**–NIP**5_h_**, and a volume of 1 mL of methanol–water (85:15, *v*/*v*) standard solutions of BPA (concentration of 7.5 μg L^−1^) were added. The tubes were sealed, and for the MIP**1_b_** and NIP**1_b_** oscillated with a thermoshaker at 25 °C for 2 h, and for the MIP**1_h_**–MIP**5_h_** and NIP**1_h_**–NIP**5_h_**, were first manually, gently stirred and then left at room temperature for 2 h. Then, the aliquots of the supernatant were used to analyze the unbound amounts of compound using LC-MS/MS (for the MIP**1_b_** and NIP**1_b_**, the tubes were first centrifuged). All measurements were carried out in triplicate. The binding capacities (*B*, μg g^−1^) of MIP**1_b_**, NIP**1_b_**, MIP**1_h_**–MIP**5_h_**, and NIP**1_h_**–NIP**5_h_** were calculated according to Equation (1):

(1) $$B=\frac{(c_{i}-c_{f})V}{m}$$

and followed by the calculation of the distribution coefficients (*Kd*, L g^−1^) for MIP**1_b_**, NIP**1_b_**, MIP**1_h_**-MIP**5_h_**, and NIP**1_h_**–NIP**5_h_** according to Equation (2):

(2) $$K_{D}=\frac{(c_{i}-c_{f})V}{c_{f}\;m}$$

where *V* represents a volume of solution (L), *c_i_* represents the initial solution concentration (μg L^−1^), *c_f_* represents the solution concentration after adsorption (μg L^−1^), and *m* represents the mass of the polymer sample (g). The imprinting factors (*IF*) were calculated according to Equation (3):

(3) $$\mathrm{IF}=\frac{K_{D}(MIP)}{K_{D}(NIP)}$$

### 2.4. Release Studies

For preliminary desorption studies, first, the soaking of MIP**1_h_** and NIP**1_h_** in the standard solution of BPA at the concentrations of 20 μg L^−1^, 200 μg L^−1^, or 1000 μg L^−1^ in the methanol–water system (85:15, *v*/*v*) was performed, according to the procedure described in Section 2.3. The aliquots of the supernatant were used to analyze the unbound amounts of compound using LC-MS/MS. Then, to study the desorption process, the solutions of BPA were replaced with 1 mL of methanol–water (85:15, *v*/*v*) for each sample. At 20, 60, and 120 min after the replacement of solutions, aliquots of the supernatant were collected for LC-MS/MS analysis. After each aliquot collection, the volumes of methanol–water (85:15, *v*/*v*) solutions were adjusted to 1 mL. All measurements were carried out in triplicate.

For the release in PBS studies, first, the soaking of MIP**1_h_** and NIP**1_h_** in the standard solution of BPA at the concentration of 200 μg L^−1^ in the methanol–water system (85:15, *v*/*v*)—in various pH values, viz. 2, 5, and 9.5—was performed, according to the procedure described in Section 2.3. The pH values of the standard solutions were adjusted with the use of 1 M aq. hydrochloric acid (for pH 2), methanol–1% aq. formic acid (for pH 5), and methanol–1% aq. ammonium hydroxide (for pH 9.5). The aliquots of the supernatant were used to analyze the unbound amounts of compound using LC-MS/MS. Then, to study the release process, the solutions of BPA were replaced with 1 mL of PBS for each sample. At 20, 60, and 120 min after the replacement of solutions, aliquots of the supernatant were collected for LC-MS/MS analysis. After each aliquot collection, the volumes of PBS solutions were adjusted to 1 mL. All measurements were carried out in triplicate. 

### 2.5. Instrumentation

Instrumental analysis was performed using an Agilent 1260 Infinity System (Agilent Technologies, Santa Clara, CA, USA), equipped with a degasser, a thermostatted column compartment, an autosampler, and a binary pump, coupled to a QTRAP 4000 hybrid triple quadrupole/linear ion trap mass spectrometer (AB Sciex, Framingham, MA, USA). The turbo ion spray source was operated in positive mode. The curtain gas, ion source gas 1, ion source gas 2, and collision gas (all high purity nitrogen) were set at 241 kPa, 414 kPa, 276 kPa, and “medium” instrument units, respectively. The ion spray voltage and source temperature were 5500 V and 600 °C, respectively. The target compounds were analyzed in multiple reaction monitoring (MRM) mode. The quantitative MRM transitions, declustering potential (DP), and collision energy (CE) for BPA were (*m*/*z*) 210/164 (DP = 66 V, CE = 18 V). Chromatographic separation was achieved with a Kinetex^®^ EVO C18 column (100 mm × 4.6 mm, 2.6 μm) from Phenomenex (Torrance, CA, USA). The column was maintained at 40 °C at a flow rate of 0.5 mL min^−1^. The mobile phase consisted of water with 0.2% acetic acid as eluent A (90%) and acetonitrile with 0.2% acetic acid as eluent B (10%). The run lasted for 5 min. The injection volume was 10 μL. 

The surface morphology analysis using scanning electron microscopy (SEM) with a Merlin FE-SEM (Zeiss, Oberkochen, Germany) was performed at the Faculty of Chemistry, University of Warsaw, Poland. The samples were Au/Pd-sputter-coated before SEM analysis. The infrared (FT-IR) spectra were recorded on Nicolet iS50 FT-IR (Thermo Fisher Scientific, Waltham, MA, USA) at the Biological and Chemical Research Centre, University of Warsaw, Poland. 

### 2.6. Theoretical Analysis

Molecular modelling methodology was performed according to previous works [19,20]. Briefly, the optimized geometries of all compounds and the so-called ESP (electrostatic potential) atomic partial charges on the atoms were obtained using the density functional theory (DFT) with B3LYP/6-311þG (d,p) hybrid functional implemented in the Gaussian 16 program [21]. Random starting models of analyzed systems were created using the Packmol software [22]. A CHARMM force field [23] was applied during molecular mechanics (MM) and molecular dynamic (MD) simulations performed using the BIOVIA Discovery Studio 2021 software package [24]. The MM process consisted of two cycles of minimization—the first using 100 steps of steepest descent, and the second using 10,000 steps of conjugate gradient—and was applied until the RMS gradient of the structure reached below 0.01 kcal mol^−1^ Å^−1^. The MD protocol contained a heating step (100 ps, time steps of 1 fs). The system was heated from 0 to 300 K. Prior to the production stage, isothermal equilibration was performed (100 ps, temperature of 300 K). The production run was conducted for 5 ns in the NVT ensemble (constant-volume/constant-temperature dynamics) at 300 K, and the coordinates were recorded every 10 ps. For the MD protocols, the Leapfrog Verlet integration and SHAKE [25] algorithms were applied. Trajectory file data generated from the NVT MD simulation have been used in all the calculations and analyses presented in this research.

The first step of the simulation process was performed to find the best monomer for synthetic procedure. Thirteen different monomer structures, viz. acrylamide, N-allylaniline, N-allylurea, 1-allylpiperazine, 2-acrylamido-2-methylpropane sulfonic acid, 2-(diethylamino)ethyl methacrylate, 1,1,1,3,3,3-hexafluoroisopropyl methacrylate, 2-hydroxyethyl methacrylate, acrylic acid, isopropenylbenzene, 2-(trifluoromethyl)acrylic acid, 4-vinylpyridine, and methacrylic acid were tested. During the studies, simple models of the pre-polymerization complexes were created from the template and the functional monomers. The boxes with the template molecule (cation of L-phenylalanine, cationic form was used because of the acidic solvent used during synthetic procedure) surrounded by six molecules of appropriate monomer molecules were prepared, and starting structures were created using Packmol. Then, the energy optimization procedure using MM was applied to obtain models of pre-polymerization systems with intermolecular interactions formed between the template and monomers. The monomer that gave the lowest complexation energy value was selected for the construction of the MIP cavity model and for polymer synthesis. The complexation energies (Δ*Ec*, kcal mol^−1^) were calculated according to Equation (4):

Δ*Ec* = *E_system_* − *E_template_* − *E_monomers_*, (4)

where *E_system_* is the potential energy of the pre-polymerization complex, *E_template_* is the potential energy of the template molecule, and *E_monomers_* is the potential energy of the monomers without the template.

In the next step of computational analysis, the model of the MIP cavity was built, constructed from the monomer (methacrylic acid) chosen in the previous step, and simulation of the BPA adsorption process on the MIP matrix was performed to analyze and define interactions responsible for molecular recognition of the analyte with MIP. The MIP cavity starting structure was obtained during the construction of the box with the template surrounded by methacrylic acid molecules, followed by the addition of the cross-linker (ethylene glycol dimethacrylate) and porogen molecules (67 molecules of toluene, 15 molecules of acetic acid, 6 molecules of trifluoroacetic acid, and 1 molecule of trifluoroacetic acid anion) using Packmol. Then, MM and MD procedures were applied. Next, the C atoms present in the monomer and cross-linker vinyl groups that were closest to each other were bonded, taking into account the fact that all molecules of the monomer and cross-linker in the studied system should create one cross-linked polymeric chain. The operation described mimicked the polymerization reaction in the synthetic process. The so-called ESP atomic partial charges were calculated for the created polymeric chain structure, and the MM and MD procedures were repeated for the system constructed from the polymeric chain, the template and the solvent, to form a specific binding site in the polymer.

The obtained model of the MIP chain was applied during the modelling of the BPA adsorption process. The template was removed, and the empty space was proposed as the computer model of recognition site. The molecule of the analyte was inserted into the MIP cavity model (replacing the template). Different forms of the analyte, viz. cationic, anionic, and zwitterion, were analyzed according to different pH values of the adsorption process. Then, the MD simulation was carried out using the dielectric constant value of the methanol–water (85:15, *v*/*v*) solution, *ε* = 36r_ij_ [26], mimicking the experimental conditions of adsorption. 

### 2.7. Cytotoxicity Tests

In order to assess the cytotoxicity of the most promising composite, MIP**1_h_**, the MTT assay was used on V79-4 and A549 cell lines. The growth media used for V79-4 and A549 cell lines were, respectively, DMEM with a 10% addition of FBS and two antibiotics: 0.1 mg mL^−1^ of streptomycin and 100 IU mL^−1^ of penicillin, as well as F-12K with a 10% addition of FBS and two antibiotics: 0.1 mg mL^−1^ of streptomycin and 100 IU mL^−1^ of penicillin. Briefly, V79-4 and A549 cells were seeded in culture media on 96-well microplates at a density of 8 × 10^3^ and 1 × 10^4^, respectively, and incubated for 48 h in controlled conditions at 36.8 °C, 5% CO_2_, and 95% humidity. At the end of the incubation, each well was examined under a microscope to confirm the 90% confluency of cells. After that, the medium was discarded and replaced with extracts of the MIP**1_h_** sample. To obtain the extracts for the experiment, samples were incubated for 24 h in the cell culture media (50 g L^−1^) with FBS concentration reduced to 5% at 36.8 °C, then gently stirred and sterilized by filtration using 0.22 µm syringe filters. The cells were exposed to different dilutions of the extracts in a twofold dilution series for 20 h at 36.8 °C, 5% CO_2_, and 95% humidity (twelve data points for each dilution). Afterwards, the extracts were removed, and the cells were washed with PBS and then treated with fresh media containing MTT (final concentration of 0.25 g L^−1^) and incubated for another 3 h. Then, cells were treated with acidified propan-2-ol (0.04 M HCl) to stop the reaction and dissolve purple formazan crystals. The absorbance of the solution in each well was colorimetrically assessed at 570 nm using the Epoch™ microplate reader (Agilent Technologies, Santa Clara, CA, USA), and the optical density (OD) values were obtained. The viability of the cells was evaluated by comparing the OD_570_ results with the mean result of the control group (cells incubated in the same conditions as the experimental group without the added MIP**1_h_** extract). The MIP**1_h_** material was considered non-cytotoxic in the tested range of concentrations if the cell viability was not reduced to below 70%.

## 3. Results and Discussion

### 3.1. Synthesis of the Materials

#### 3.1.1. Theoretical Choice of Functional Monomer

Firstly, computational modelling was used to choose the monomer that would be able to form the polymer with the highest binding affinity towards the analyte—BPA. To simplify and make the synthetic and simulation procedures cost- and time-effective, simple models of pre-polymerization complexes consisting of monomers and template molecules were constructed. As the template, L-phenylalanine (structure resembling the analyte) was used in experimental and theoretical studies to avoid the analyte bleeding phenomenon. It was expected that the functional monomer which showed the lowest complexation energy towards the template should produce the polymer with the highest affinity [27]. Potential functional monomers that could possibly form complexes with the template due to non-covalent interactions were tested for their complexation energy. Thirteen different molecules possessing polymerizable groups and residues that could interact with the template via non-covalent interactions were studied. The results are presented in Table 2.

As one can see, two acidic monomers, methacrylic (Δ*Ec* = −75.13 kcal mol^−1^) and acrylic acid (Δ*Ec* = −75.07 kcal mol^−1^), were identified as the most promising monomers that could form strong interactions with the template (Table 2). The ability of the described two monomers to create a stable pre-polymerization complex was mainly associated with the formation of hydrogen bonds between the monomer –COOH group and the template –COOH or –NH_3_^+^ groups. 

#### 3.1.2. Verification of Effectiveness of MIP with Bulk Polymer 

**Figure 1:**
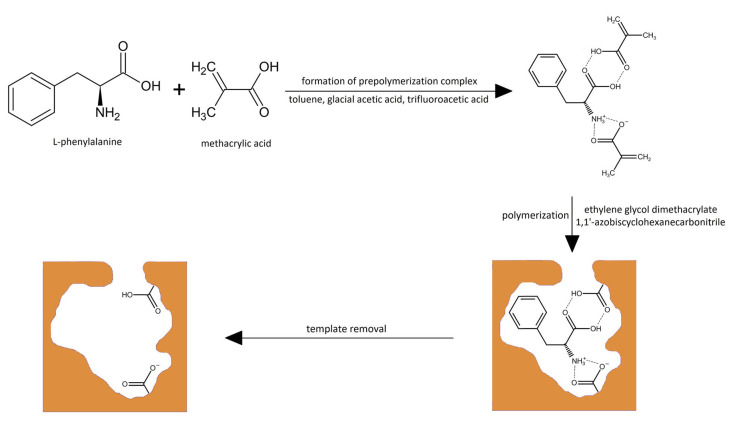
Scheme of MIP synthesis with a matrix based on L-phenylalanine as the template, methacrylic acid as the monomer, and ethylene glycol dimethacrylate as the cross-linker.

In the next step of our investigations, the experimental verification of results from the theoretical analysis was performed. For that purpose, the functional monomer that was characterized by the lowest energy of the stabilization of the pre-polymerization complex with the L-phenylalanine template was chosen, viz. methacrylic acid, and the synthesis of the MIP was carried out in the presence of ethylene glycol dimethacrylate as the cross-linking agent (the scheme is presented in Figure 1). Here, the bulk material was prepared, and its capability to adsorb the BPA was evaluated in order to assess the effectiveness of the MIP system when compared to NIP, which was also synthesized. The effectiveness of the MIP was determined based on the binding capacity (Equation (1)), distribution coefficient (Equation (2)), and the specificity expressed by the imprinting factor (Equation (3)). The results are provided in Table 3.

As could be seen, the MIP prepared from methacrylic acid and ethylene glycol dimethacrylate was characterized by a nearly three-and-a-half-fold higher binding capacity of BPA than NIP. The comparison of distribution coefficients revealed high specificity with *IF* equal to 4.36. Although the capability of a methacrylic-acid-based MIP imprinted by L-phenylalanine was evaluated previously [28], to the best our knowledge, the investigations of such a system for the specific adsorption of BPA has not been executed. Thus, it could be concluded that the MIP system synthesized from the methacrylic acid and ethylene glycol dimethacrylate in the presence of L-phenylalanine was effective for the adsorption of BPA. It should be underlined that the experimental results also confirmed the findings of the theoretical analysis, which could be used as the predictable and low-cost tools for the preselection of functional monomers for the MIP’s synthesis. 

#### 3.1.3. Theoretical Explanation of Interactions in the MIP Cavity

**Figure 2:**
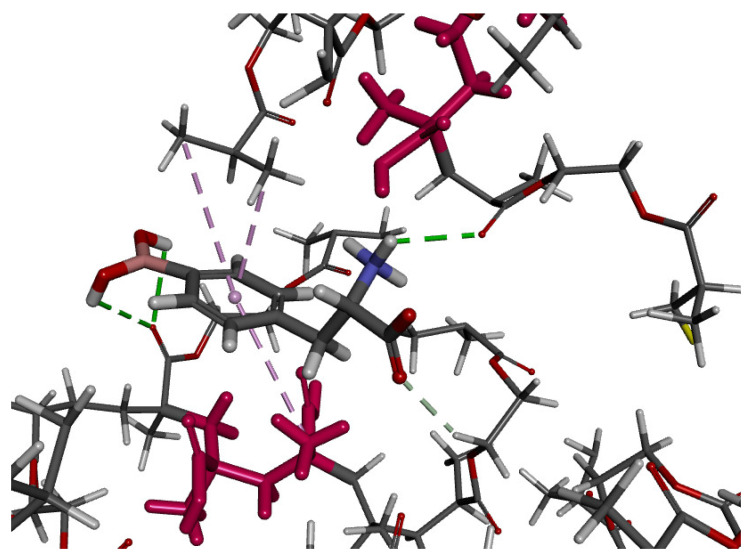
The cavity of MIP with the analyte inside. Monomer residues are shown as the pink molecules. The classical hydrogen bonds are shown as the green dashed lines, the non-classical hydrogen bonds are shown as the gray dashed lines, and the hydrophobic interactions are shown as the pink dashed lines.

On the basis of monomer selection results, the model of the polymer cavity was prepared, and the simulation of BPA adsorption was performed to identify the interactions responsible for the molecular recognition process on the MIP matrix. Different forms of the analyte were analyzed. In the optimized system with a zwitterionic form of BPA present in acidic-neutral solutions, three classical hydrogen bonds were observed: two between the H atoms of the –B(OH)_2_ group from the analyte molecule and the O atom of the cross-linker residue (length of 2.60 and 3.00 Å) and one between the H atom of the –NH_3_^+^ group from the analyte and the O atom of the cross-linker residue (length of 2.47 Å). Additionally, one non-classical hydrogen bond was formed between the O atom of the –COO^−^ group from the analyte and the H atom of the –CH_2_– group from the cross-linker residue (length of 2.38 Å), and three hydrophobic π-alkyl-type interactions were present between the aromatic ring of the analyte and the –CH_3_ groups of the cross-linker and monomer residues (lengths of 3.78, 4.58, and 4.68 Å, Figure 2). We observed that all three functional groups of the analyte (–B(OH)_2_, –COO^−^, and –NH_3_^+^) were involved in interactions with the polymeric matrix, and those interactions were important in the molecular recognition mechanism observed in the studied MIP system.

**Figure 3:**
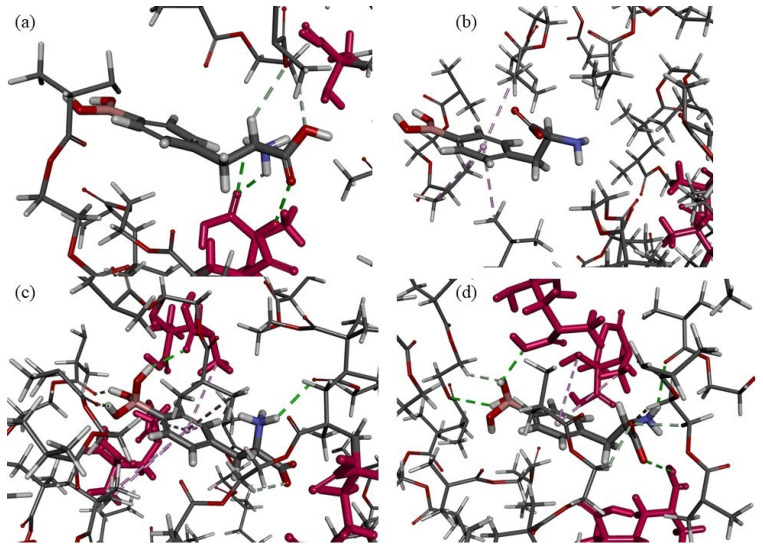
The cavity of MIP with different species of analyte inside: (**a**) cationic form; (**b**) anionic form with –COO^−^ group; (**c**) zwitterion with –B(OH)_3_^−^ group; (**d**) anion with –COO^−^ and –B(OH)_3_^−^ groups. Monomer residues are shown as the pink molecules. The classical hydrogen bonds are shown as the green dashed lines, the non-classical hydrogen bonds are shown as the gray dashed lines, and the hydrophobic interactions are shown as the pink dashed lines.

In acidic solutions, the main form of BPA is a cationic form with a protonated amine group. In the simulated system with analyte cation, three classical hydrogen bonds were formed: two between the H atoms of the –NH_3_^+^ group from the analyte and the O atom of the –COOH group from the monomer residue (length of 2.12 and 2.63 Å) and one between the O atom of the –COOH group from the analyte and the H atom of the –COOH group from the monomer residue (length of 2.37 Å). Also, two non-classical hydrogen bonds were observed between the O atom of the –COOH group from the analyte and the H atom of the –CH_2_– group from the cross-linker residue (length of 2.76 Å), as well as between the H atom of the –CH– group from the analyte and the O atom from the cross-linker (length of 2.71 Å, Figure 3a). Only two functional groups (–COOH and –NH_3_^+^) of BPA participated in the interactions’ formations during the simulation of the adsorption process of the analyte cationic form on MIP material. No interactions of the analyte –B(OH)_2_ group with a polymeric matrix might be one of the reasons for a low MIP binding capacity of BPA from an acidic solution. 

When the pH of an analyte solution is basic, anionic species of the analyte are present. Three different anions could be created: one with the COO^−^ group or zwitterion with the B(OH)_3_^−^ group (charge of species is minus one) or one with both above mentioned groups (charge of form is minus two) [29]. In the system simulating the adsorption of an analyte anion with COO^−^ group, we observed only three hydrophobic π-alkyl-type interactions between the aromatic ring of the analyte and the –CH_3_ groups of the cross-linker residues (lengths of 4.05, 4.10, and 4.89 Å, Figure 3b). No interactions were formed with the participation of the main functional groups of BPA, viz. –NH_2_, –COO^−^, and –B(OH)_2_. On the other hand, when the systems modelling the adsorption process of two other anions (zwitterion with B(OH)_3_^−^ group and the species with both COO^−^ and B(OH)_3_^−^ groups) were analyzed, we could find interactions between all functional groups of the analyte and polymeric matrix. In the model of the B(OH)_3_^−^ zwitterion adsorption, four classical hydrogen bonds were formed: three between the H atoms of the –B(OH)_3_^−^ group from the analyte molecule and the O atoms of the monomer or cross-linker residues (lengths of 2.19, 2.16, and 2.33 Å) and one between the H atom of the –NH_3_^+^ group from the analyte and the O atom of the cross-linker residue (length of 2.70 Å). Additionally, one non-classical hydrogen bond was formed between the O atom of the –COO^−^ group from the analyte and the H atom of the –CH_2_– group from the cross-linker residue (length of 2.58 Å), and seven hydrophobic π-alkyl-type interactions were present between the aromatic ring of the analyte and the –CH_3_ groups of the cross-linker and monomer residues (length between 4.41 and 5.49 Å, Figure 3c). In the optimized system simulating the adsorption of an anion with two minus charges, we observed four classical hydrogen bonds: two between the H atoms of the –B(OH)_3_^−^ group from the analyte molecule and the O atoms of the monomer or cross-linker residues (lengths of 2.22 and 2.92 Å), one between the H atom of the –NH_2_ group from the analyte and the O atom of the cross-linker residue (length of 2.55 Å), and one between the O atom of the –COO^−^ group from the analyte and the H atom of the –COOH group from the monomer residue (length of 2.82 Å). Also, four non-classical hydrogen bonds were formed: one between the O atom of B(OH)_3_^−^ group from the analyte and the H atom of the –CH_2_– group from the cross-linker residue (length of 2.43 Å) and three between the O atom of the –COO^−^ group from the analyte and the H atoms of the –CH_2_– groups from the cross-linker residues (lengths of 2.71, 2.75, and 2.80 Å). Moreover, two π-alkyl-type interactions were present between the aromatic ring of the analyte and the –CH_3_ groups of the monomer residues (lengths of 4.66 and 5.39 Å, Figure 3d). The types of interactions and functional groups of the analytes involved in the adsorption process on the MIP matrix in computational studies were similar when the zwitterion form and two anionic forms of BPA were analyzed. The coexistence of different anionic forms of the analyte in basic solution that could interact with the MIP in different ways might result in a lower specificity of MIP towards analyte in basic solution. 

#### 3.1.4. Fabrication of Honeycomb-like Polymer and Adsorption Capabilities 

In the following step of the investigation, we aimed to construct the MIP with enhanced binding capacity. It is known from the literature survey that more sophisticated D- and L-phenylalanine imprinted systems, such as MIP conjugates with gold nanoparticles [30] or MIPs synthesized in the presence of chiral ionic liquids [31], could affect the binding capacity of the resulted materials, being promising sorbents for separation purposes. However, it should be emphasized that the application of those systems for BNCT could be limited due to biosafety reasons. Thus, we concluded that the use of well-known biocompatible components for the MIP synthesis will be beneficiary [32]. The previously optimized composition system based on methacrylic acid and ethylene glycol dimethacrylate was used in the synthesis of honeycomb-like structures with an extended surface area (the scheme is presented in Figure 1). The honeycomb-like structures have found applications in many diverse fields such as architecture or mechanical engineering, but most importantly, they are highly utilized in biomedicine [33]. 

In the fabrication process of the honeycomb-like structure, the siloxane particles that were functionalized with MPS were used to provide a support for the polymer matrix prior to the etching of siloxane material. It allowed us to obtain the structural periodic systems of a regular nature and an extended external surface. Moreover, we have expanded our investigation for the analysis of the cross-linker effect on the binding capacity of honeycomb-like MIPs towards BPA. For that purpose, five MIPs were composed from methacrylic acid and ethylene glycol dimethacrylate as well as four other cross-linkers, viz. poly(ethylene) glycol dimethacrylate, trimethylolpropane trimethacrylate, triethylene glycol dimethacrylate, and divinylbenzene. Although the last cross-linker cannot be considered as a biocompatible reagent, it was very interesting for us to explore the effect of the rigid molecular structure of divinylbenzene on the formation of honeycomb-like structure. The results of the binding capacities, distribution coefficients, and *IF*s are presented in Table 3. 

As can be seen, the honeycomb-like structure of MIP**1_h_** was characterized by the higher binding capacity of BPA when compared to bulk MIP**1_b_**. It could be explained by the higher accessibility of the adsorption sites to the target molecule. However, simultaneously, we observed a decrease in the specificity of MIP**1_h_** when compared to bulk MIP**1_b_** due to significantly increased adsorption on the NIP**1_h_**. The specificity was reduced by more than double when compared to bulk material. We could suppose that the higher accessibility of the extended surface of the honeycomb-like structure resulted also with higher non-specific adsorption. This phenomenon was confirmed in the next analyzed systems that were characterized by higher non-specific adsorption of non-imprinted materials, and the specificities of those systems were unsatisfactory. The MIP**2_h_**, MIP**3_h_**, MIP**4_h_**, and MIP**5_h_** were characterized by the moderate binding capacity with a decreasing trend. As can be expected, the MIP**5_h_** synthesized from the rigid cross-linker, divinylbenzene, was characterized by the lowest binding capacity of BPA among all polymers.

### 3.2. Characterization of Honeycomb-like Polymer

#### 3.2.1. Morphological Studies

**Figure 4:**
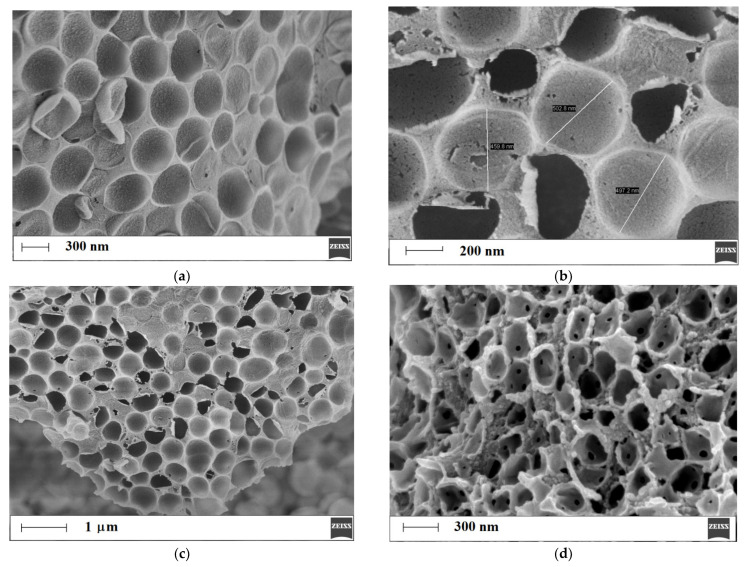

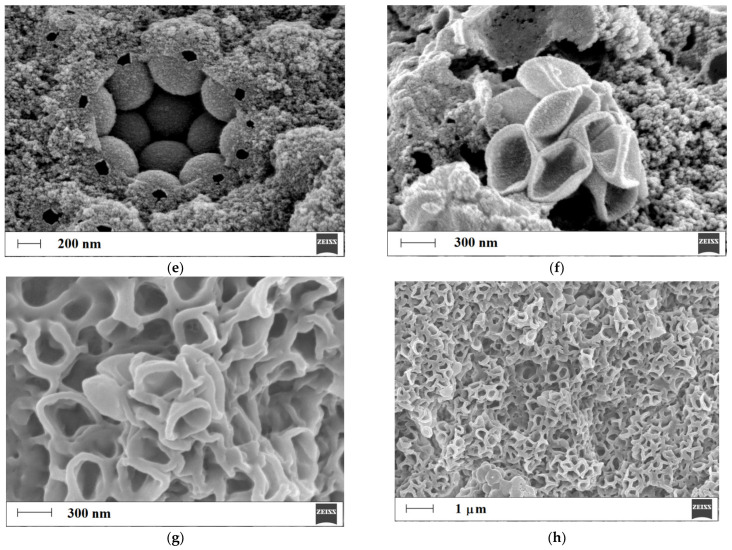
Micrographs of honeycomb-like structures of MIP**1_h_**/NIP**1_h_** (**a**–**f**) and MIP**2_h_**/NIP**2_h_** (**g**,**h**).

In order to confirm the formation of the honeycomb-like structure, SEM analysis was employed, and the micrographs of the copolymers of methacrylic acid-co-ethylene glycol dimethacrylate, MIP**1_h_**/NIP**1_h_** (Figure 4a–f), and methacrylic acid-co-poly(ethylene) glycol dimethacrylate, MIP**2_h_**/NIP**2_h_** (Figure 4g,h), were analyzed. 

As can be seen, the micrographs of MIP**1_h_**/NIP**1_h_** in high magnification (Figure 4a–c) revealed an ordered porous, honeycomb-like structure. It was clearly detected that the polymer formed over the siloxane support possessed periodic intervals that were created after the etching of functionalized siloxane particles. We have found that internal layers of the polymer were more homogeneous (Figure 4a–c) than external layers (Figure 4d), but the honeycomb-like structure was still maintained. The diameter of the periodic structure of the honeycomb-like element was equal to 487 ± 23 nm (Figure 4b). The external layer revealed multiple holes (Figure 4d), and their nature was investigated more deeply. We have found nestle-like regions with multiple polymer entities that were etched (Figure 4e). We could suppose that these holes in the polymeric spherical particles originated from the breaks in the polymeric layer, allowing aqueous hydrofluoric acid to be transferred into the internal siloxane core of the particles. These breaks could be a result of the conglomeration of siloxane functionalized particles during the polymerization process. Moreover, the effectiveness of the etching of internal siloxane cores was proved. We have also observed sunken, hollow-like structures (Figure 4f). As can also be seen, the micrographs of MIP**2_h_**/NIP**2_h_** in high magnification (Figure 4g,h) revealed an ordered, porous, honeycomb-like structure. However, the character of this structure differed when compared to the honeycomb-like structure of MIP**1_h_**/NIP**1_h_**. The remaining polymer skeleton after etching could be characterized by thicker layers with a smoother surface. We could only suppose that the higher viscosity of the poly(ethylene) glycol dimethacrylate component of the pre-polymerization mixture (approx. 70 mPa s) when compared to the ethylene glycol dimethacrylate (approx. 2 mPa s) component was responsible for the morphology of MIP**2_h_**/NIP**2_h_** [34,35].

#### 3.2.2. Structural Analysis

**Figure 5:**
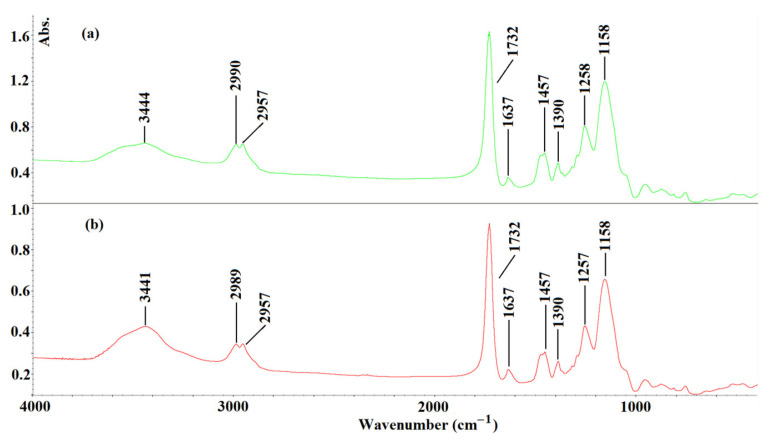
FT-IR spectra of MIP**1_h_** after synthesis and extraction of template ((**a**), green line), and MIP**1_h_** after soaked in the standard solution of BPA and dried ((**b**), red line).

The FT-IR analysis was employed to prove the structure of the obtained MIP**1_h_** (Figure 5a). The MIP**1_h_** was analyzed after the synthesis and the extraction of the template molecule of L-phenylalanine. To reveal the predicted interactions between BPA and the MIP**1_h_**, the FT-IR analysis was extended to the sample of a polymer soaked in the standard solution of BPA and dried (Figure 5b). 

As can be seen, the characteristic vibration peaks, derived from structural fragments of MIP**1_h_** after the synthesis and extraction of the template, could be assigned as follows: at 3444 cm^−1^ (broad), the –OH stretching vibrations; at 2990 and at 2957 cm^−1^, the stretching vibration of the *sp*^2^ and *sp*^3^ bonds of the –CH from the cross-linker; at 1732 cm^−1^, the –C=O stretching vibration; at 1637 cm^−1^, the stretching of –C=C bonds; at 1457 cm^−1^, the stretching of –CH_2_–CH_2_; at 1390 cm^−1^, the stretching of –CH_3_; at 1258 cm^−1^ and 1158 cm^−1^, the C–O–C stretching vibrations. A very similar pattern of the FT-IR was observed for the MIP**1_h_** after being soaked in the standard solution of BPA and dried. The only difference noted between the two spectra that were recorded was related to the region of the –OH stretching vibrations. The shift of the broad peak maximum from 3444 cm^−1^ to 3441 cm^−1^ could be explained by the presence of –B(OH)_2_ group in the BPA molecule and the possible existence of hydrogen bonds between the BPA and the residues of the methacrylic acid in the MIP**1_h_**. 

### 3.3. Release Studies

**Figure 6:**
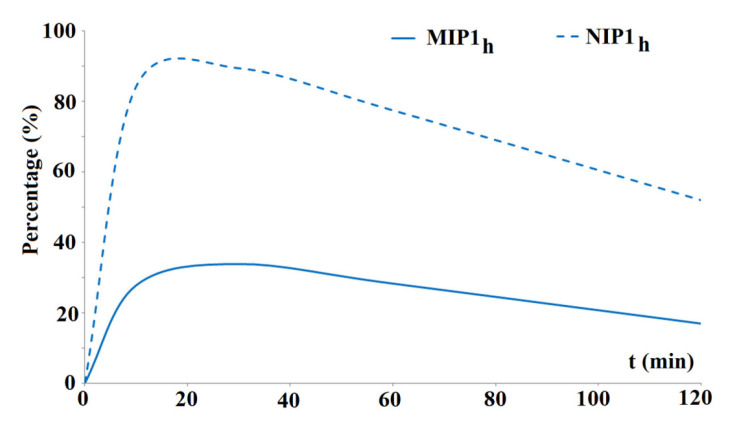
Desorption profiles of BPA from MIP**1_h_** and NIP**1_h_** to the methanol–water system (85:15, *v*/*v*).

The preliminary desorption studies of BPA were conducted after soaking MIP**1_h_** and NIP**1_h_** in the standard solution of BPA at the concentrations of 20 μg L^−1^, 200 μg L^−1^, or 1000 μg L^−1^ in the methanol–water system (85:15, *v*/*v*). Firstly, the following binding capacities were obtained for MIP**1_h_**: 86.5 ± 3.3 ng g^−1^, 715.0 ± 6.1 ng g^−1^, 6970 ± 112 ng g^−1^, respectively, and for NIP**1_h_**: 44 ± 0.10 ng g^−1^, 370 ± 5.5 ng g^−1^, 4155 ± 31 ng g^−1^, respectively. The calculated *IFs* were as follows: 1.97 (for a concentration of 20 μg L^−1^), 1.93 (for a concentration of 200 μg L^−1^), and 1.68 (for a concentration of 1000 μg L^−1^). The results confirmed the increase in the binding capacity of MIP**1_h_** and NIP**1_h_** with the increased concentration of the standard solution of BPA. The specificity remained similar in the concentration range of BPA between 20–200 μg L^−1^ but slightly decreased in a higher concentration of 1000 μg L^−1^. Thus, to evaluate the desorption capabilities of MIP**1_h_** and NIP**1_h_**, the samples loaded with the concentration of 1000 μg L^−1^ were taken for analysis. Figure 6 presents the desorption profiles, expressed as the percentage of the loaded amount of BPA from MIP**1_h_** and NIP**1_h_** to the methanol–water system (85:15, *v*/*v*) in time. 

As could be seen, the significant difference in the desorption of BPA in the methanol–water system (85:15, *v*/*v*) from MIP**1_h_** and NIP**1_h_** was observed in time. The desorption was fast and proceeded mostly during the first half hour of the experiment with the values of 33.82% for MIP**1_h_** and 89.46% for NIP**1_h_**. The lower amount of BPA that was desorbed from MIP**1_h_** when compared to NIP**1_h_** could be explained by the presence of the surface modified regions in the polymeric network that were responsible for the adsorption/desorption phenomenon (the so-called ‘tumbling effect’) that slowed down the desorption process of the BPA.

In the next step, the release profiles from the model PBS were performed in various pH values, viz. 2, 5, and 9.5. It is known that the BPA could exist in various ionic forms that depend on the pH of the system. The p*K*_a_ values are as follows: 2.46 (p*K*_a_l_), 8.46 (p*K*_a_2_), and 9.76 (p*K*_a_3_) [29]. Firstly, the binding capacities in the above-mentioned pH values of the BPA from the adjusted methanol–water (85:15, *v*/*v*) solution were analyzed. The binding capacities were the following for the pH 2: 370.0 ± 1.0 ng g^−1^ for MIP**1_h_** and 310 ± 15 ng g^−1^ for NIP**1_h_**; for the pH 5: 1905 ± 14 ng g^−1^ for MIP**1_h_** and 1105 ± 16 ng g^−1^ for NIP**1_h_**; for the pH 9.5: 1345 ± 53 ng g^−1^ for MIP**1_h_** and 1670 ± 46 ng g^−1^ for NIP**1_h_**. The specificity expressed as the *IFs* were the following: 1.19 for pH 2, 1.72 for pH 5, and 0.81 for pH 9.5. The results suggested that the binding capacity of BPA on the MIP**1_h_** increased nearly fivefold with the increase in the pH of the adsorption system from pH 2 to pH 5 and then was nearly halved in pH 9.5. In contrast, the binding capacity of BPA on the NIP**1_h_** increased constantly from an acidic to basic environment of the loading system. The remarkable difference in pH 9.5 was responsible for the lack of specificity of MIP**1_h_**.

**Figure 7:**
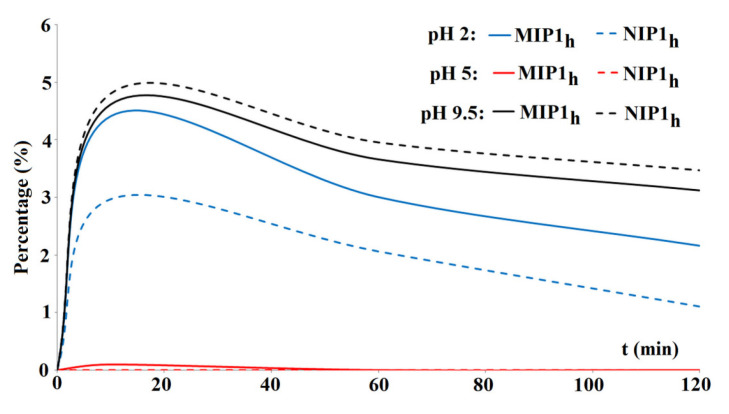
Release profiles of BPA from MIP**1_h_** and NIP**1_h_** to PBS.

The release profiles of BPA from MIP**1_h_** and NIP**1_h_** expressed as the percentage of the loaded amount are presented in Figure 7. For the purpose of this experiment, the loading standard solution of a concentration of 200 μg L^−1^ was applied to emphasize the specificity. 

As can be seen, the percentage of the BPA that was released from the MIP**1_h_** and NIP**1_h_** was low (between 0% and 4.81%), and the peak occurred within the first 20 min of the experiment. In the sample adjusted to pH 2, the released amount of the BPA from MIP**1_h_** was higher than from NIP**1_h_**. It could be explained by the protonation of the amine group in the BPA molecule and the competitive protonation of the carboxylic residues in the MIP**1_h_**. In the sample adjusted to pH 5, the released amount of the BPA from MIP**1_h_** and NIP**1_h_** was very small. The zwitterion insoluble form of the BPA at pH 5 could explain the result. In the sample adjusted to pH 9.5, the released amount of the BPA from MIP**1_h_** was lower than from NIP**1_h_**. The additional interactions of anionic regions of –B(OH)_3_^−^ with the polymer network could explain the decrease in release ratio. 

### 3.4. Cytotoxicity Tests

**Figure 8:**
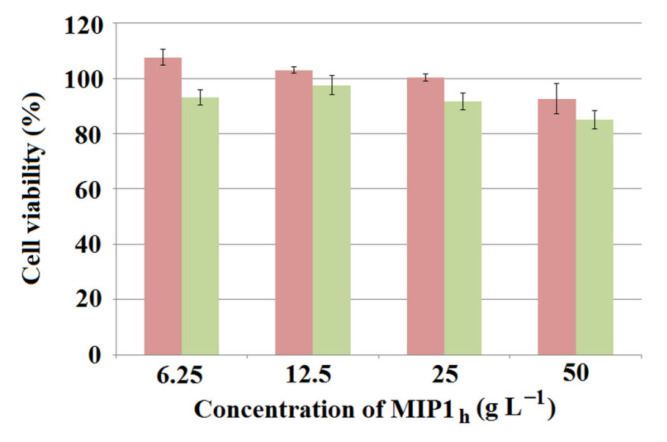
The MTT test results on A549 (red bars) and V79-4 (green bars) cell lines, obtained from MIP**1_h_** extracts within the whole range of examined concentrations.

Viability experiments using the MTT assay were conducted using two different cell lines (cancerous and normal) to assess the initial safety of the synthesized honeycomb-like polymer with the most promising properties, MIP**1_h_**. The V79-4 (normal) and A549 (cancerous) cells were exposed to four different concentrations of MIP**1_h_** extracts for 20 h at 36.8 °C. The results presented in Figure 8 show a high cell viability and negligible cytotoxicity within the tested range of concentrations, suggesting good biocompatibility of the material under examined conditions and a potential for in vivo applications. For the V79-4 cell line, the viability was above 80% for the entire range of concentrations, and for the A549 cell line, it was above 90%. This is in accordance with the assumptions based on the composition of synthesized material—other MIPs for biomedical applications synthesized from methacrylic acid and ethylene glycol dimethacrylate have been reported as being low-toxic [36,37].

## 4. Conclusions

A honeycomb-like MIP composed from methacrylic acid copolymerized with ethylene glycol dimethacrylate was successfully synthesized for specific recognition towards BPA. Theoretical analysis was employed to choose the most suitable monomer, which was further confirmed by experiments on bulk polymer. The specificity of a honeycomb-like MIP compared to a bulk polymer of the same matrix was reduced due to the higher non-specific adsorption caused by higher accessibility of the extended surface of the structure. The increased binding surface was also reflected in a honeycomb-like MIP’s higher binding capacity of BPA when compared to a bulk polymer, proving honeycomb-like MIPs a more attractive material for further investigations related to the delivery of boron to the target site. The adsorption properties in different pH were further explained by the theoretical studies. The ordered, porous morphology of honeycomb-like MIPs was confirmed with SEM, and its structure was confirmed by FT-IR, proving that the material of the desired structure was obtained. The release studies in the model PBS revealed that no more than 4.81% of BPA is released from the MIP within two hours, which is a significantly beneficial result when considering the application in BNCT, meaning that the entire composite could be delivered intact to the target site. Cytotoxicity towards A549 and V79-4 cell lines within the tested range of concentrations was considered very low, suggesting the safety of the synthesized MIP for potential in vivo applications. In order to provide a therapeutic dose of boron, the binding capacity of the synthesized material still has to be improved. However, due to the delivery of BPA with a MIP system, and based on the study by Nomoto et al. on a different polymeric delivery system [8], it can be expected that the cellular internalization mechanism will change, and thus, we can assume that the accumulation in tumor cells will be more efficient than in the case of the currently used BPA–fructose complex. To the best of our knowledge, this study presents the first results of a MIP design for boron-containing molecule delivery considered for BNCT application.

## Figures and Tables

**Table 1:** Amounts of SiO_2_@MPS and cross-linkers used in the polymerization of 172.6 mg (2 mmol) of methacrylic acid in the presence of L-phenylalanine, 57.8 mg (0.35 mmol), and 20 mg of 1,1′-azobiscyclohexanecarbonitrile as the initiator in a mixture of toluene (2.5 mL), glacial acetic acid (0.3 mL), and trifluoroacetic acid (0.2 mL).

Code of MIP	Cross-Linker (g, mmol)	SiO_2_@MPS (mg)
MIP**1_b_**	ethylene glycol dimethacrylate (**1**), 1.98, 10	without
MIP**1_h_**	ethylene glycol dimethacrylate (**1**), 1.98, 10	1000
MIP**2_h_**	poly(ethylene) glycol dimethacrylate (**2**), 5.50, 10	1000
MIP**3_h_**	trimethylolpropane trimethacrylate (**3**), 3.38, 10	1000
MIP**4_h_**	triethylene glycol dimethacrylate (**4**), 2.86, 10	1000
MIP**5_h_**	divinylbenzene (**5**), 1.30, 10	1000

**Table 2:** Screening of functional monomers on their interaction with L-phenylalanine.

Functional Monomer	Complexation Energy (∆*Ec*, kcal mol^−1^)
Methacrylic acid	−75.13
Acrylic acid	−75.07
2-(Diethylamino)ethyl methacrylate	−72.00
2-Hydroxyethyl methacrylate	−68.26
N-Allylurea	−61.37
Acrylamide	−60.62
1,1,1,3,3,3-Hexafluoroisopropyl methacrylate	−59.98
4-Vinylpyridine	−57.41
2-Acrylamido-2-methylpropane sulfonic acid	−56.55
N-Allylaniline	−49.63
2-(Trifluoromethyl)acrylic acid	−48.63
1-Allylpiperazine	−40.91
Isopropenylbenzene	−33.62

**Table 3:** Binding capacities of BPA on MIP**1_b_**–NIP**1_b_** and MIP**1_h_**–MIP**5_h_**/NIP**1_h_**–NIP**5_h_** (conc. 7.5 μg L^−1^, n = 3), *Kd* and *IF*s.

No. of Polymer	Binding Capacities ± S.D. (*B*, ng g^−1^)		Distribution Ratio (*Kd*, L g^−1^)		*IF*
	MIP	NIP	MIP	NIP	
**1**_b_	269 ± 20	79.42 ± 0.26	0.041	0.009	4.36
**1**_h_	330.4 ± 4.6	197.4 ± 1.9	0.055	0.027	2.04
**2**_h_	225 ± 28	175 ± 24	0.032	0.023	1.38
**3**_h_	208 ± 11	299 ± 47	0.029	0.047	0.61
**4**_h_	176.9 ± 1.8	249 ± 18	0.023	0.036	0.64
**5**_h_	130.4 ± 3.2	254 ± 34	0.016	0.037	0.44

## Data Availability

The data presented in this study are available on request form corresponding author.
